# Application of single-cell RNA sequencing methodologies in understanding haematopoiesis and immunology

**DOI:** 10.1042/EBC20180072

**Published:** 2019-06-11

**Authors:** Anna M. Ranzoni, Paulina M. Strzelecka, Ana Cvejic

**Affiliations:** 1 Department of Haematology, University of Cambridge, Cambridge CB2 0XY, U.K.; 2 Wellcome Trust Sanger Institute, Wellcome Genome Campus, Hinxton CB10 1SA, U.K.; 3 Wellcome Trust - Medical Research Council Stem Cell Institute, Cambridge CB2 1QR, U.K.

**Keywords:** haematopoiesis, immunology, Single-cell RNA sequencing

## Abstract

The blood and immune system are characterised by utmost diversity in its cellular components. This heterogeneity can solely be resolved with the application of single-cell technologies that enable precise examination of cell-to-cell variation. Single-cell transcriptomics is continuously pushing forward our understanding of processes driving haematopoiesis and immune responses in physiological settings as well as in disease. Remarkably, in the last five years, a number of studies involving single-cell RNA sequencing (scRNA-seq) allowed the discovery of new immune cell types and revealed that haematopoiesis is a continuous rather than a stepwise process, thus challenging the classical haematopoietic lineage tree model. This review summarises the most recent studies which applied scRNA-seq to answer outstanding questions in the fields of haematology and immunology and discusses the present challenges and future directions.

## Introduction

Until the last decade, blood and immune cells were characterised by researchers using a ‘bulk’ approach, which involves studying a cell type at the population level while considering the average measure of a particular parameter in a population as representative of all the individual cells. This approach has been invaluable to unfold and characterise cell types crucial for the development of diseases. However, it has become clear that tissues conceal a much higher cellular heterogeneity than previously expected. Indeed, the average measure of a particular feature within a population is often not a representative of any of the individual cell [1]. Cell populations that seem homogeneous, in terms of expression of cell surface markers, comprise many different cell states and hide cell-to-cell variations that can have significant effects on cell function [2,3]. The transcriptome changes very rapidly in response to environmental changes and is therefore a good reflection of the particular state of a cell. One example supporting this is the observed changes in gene expression related to the cell-cycle phase [4]. Therefore, the interrogation of the whole transcriptome allows a completely unbiased analysis of a cell’s state.

Since the first report of profiling of a single-cell entire transcriptome in 2009 [5], single-cell RNA sequencing (scRNA-seq) has progressed rapidly. Several platforms have now been developed and as a result, the cost has substantially dropped. All the available methods rely on the first step in which RNA is converted into cDNA, followed by an amplification step in order to obtain sufficient amount of DNA for sequencing. Currently available platforms are divided into two categories based on the downstream methods: droplet-based (Drop-seq [6], inDrop [7], 10x Chromium Genomics [8], Seq-well [9]) and plate-based (e.g. STRT-seq [10], SmartSeq [11], SmartSeq2 [12] and MARS-Seq [13]). Plate-based approaches, combined with multiplexing, enable the interrogation of hundreds of cells, achieving an average 100 000/4 000 000 reads per cell, which are enough for the detection of lowly expressed genes such as transcription factors. Droplet-based methods on the other hand can analyse several thousand individual cells, albeit achieving an average lower numbers of reads per cell (20 000/200 000 reads per cell) [14].

## Rethinking haematopoiesis with scRNA-seq

**Figure 1:**
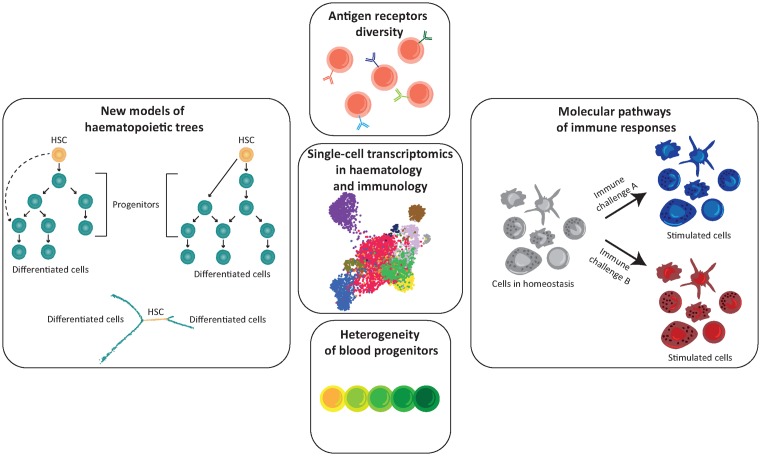
Applications of single-cell transcriptomics in haematology and immunology Advances in scRNA-seq contributed to redefining the classical haematopoietic differentiation tree and dissected the heterogeneity of blood progenitors compartment. It is now possible to study antigen receptor diversity at single-cell level, thus to better understand clonal expansion of adaptive lymphocytes and discover novel immune cell type/states related to immunological challenges.

Blood formation has been traditionally described as a stepwise process beginning from undifferentiated haematopoietic stem cells (HSCs) which undergo a series of restrictions in their lineage potential passing through a hierarchy of progenitors. This hierarchical structure is commonly depicted as a lineage tree, with HSCs at the top and progressively restricted progenitors at the nodes of branches that end with fully differentiated blood cells. This classic model was built based on the characterisation of the lineage output of cell populations defined by the expression of combinations of cell surface markers. However, it is now clear that seemingly homogeneous progenitor populations, based on phenotypic markers, are very heterogeneous in terms of cell state and potential for self-renewal and differentiation. ScRNA-seq has been extensively used in the last five years to dissect the progenitor compartment of haematopoietic tissues thanks to its unbiased approach and its sensitivity in detecting rare cell populations that would be otherwise overlooked by traditional bulk approaches [15]. Here, we report a concise account of some of the most recent studies that used scRNA-seq to address the heterogeneity of the haematopoietic progenitor compartment and shifted our understanding of the blood lineage tree structure (Figure 1). In addition, we would like to draw reader’s attention to a number of other reviews that covered this topic in recent years [16–18].

### Resolving heterogeneity of blood progenitors

A number of studies using single-cell transplantations with genetically modified mice and barcoding approaches demonstrated for the first time that haematopoietic progenitor populations are much more heterogeneous than previously suggested. These studies pointed out that phenotypic HSC populations also contain unipotent myeloid lineage-restricted progenitors, that megakaryocyte-erythroid progenitors (MEP) can arise directly from HSCs [19] and suggested the existence of platelet-biased HSCs [20]. It was also reported that lymphoid multipotent progenitors, as defined by surface markers, are a highly heterogeneous population in terms of the lineage output, containing combinations or lymphoid, myeloid and dendritic-restricted cells [21].

The application of scRNA-seq further confirmed progenitor heterogeneity and suggested the existence of unipotent populations in the pool of progenitors that were thought to be multipotent. In 2015, a study used the QUARTZ-seq protocol to characterise single phenotypic mouse HSCs and reported that the multipotent compartment, defined on the basis of cell surface markers, contained also unilineage megakaryocyte-committed progenitors [22]. The same year, MARS-seq was performed on c-Kit+ Sca1-lineage (Lin)-myeloid progenitors from mouse bone marrow and showed that granulocyte-monocyte progenitor (GMP) and common myeloid progenitor (CMP) populations together can be divided into seven main progenitor subgroups and no progenitors showed mixed state [2]. Cord blood has attracted interest in recent years after a study from Notta et al. [23] demonstrated that it contains a greater proportion of multi- and oligopotent cells compared with adult bone marrow. Using scRNA-seq and *in vitro* cultures, in addition to *in vivo* transplantation assays, this study provided the first evidence that distinct road maps of differentiation exist in the human blood system during development. In particular, it showed that in adults the stem cell compartment only is multipotent and capable of megakaryocyte/erythroid differentiation, while progenitors are mainly unipotent. This is in contrast with prenatal development, in which the megakaryocyte/erythroid potential is conserved throughout the haematopoietic tree hierarchy [23]. Drop-seq was used to sequence the transcriptome of 20 000 haematopoietic progenitors from healthy human cord blood, defined by the combination of cell-surface markers. Early progenitors formed a heterogeneous population and showed a signature of multiple lineage fates. Additionally, data suggested that progenitors could take separate trajectories to differentiate into myeloid cells; in particular, granulocytes shared early transcriptional programmes with the erythroid differentiation path [24]. A study combining scRNA-seq using the CEL-seq2 platform, functional analysis, methylome analysis and expression of 40 proteins on human cord blood cells expressing progenitor markers and CD49f, which are considered to be the most primitive progenitors, showed a striking heterogeneity in their molecular landscape and repopulating capacity following transplantation in mice [25]. Simultaneously, this strategy allowed the identification of a subset of progenitors expressing CD33 and CD90 that showed the highest long-term repopulating ability in mice. Another recent study looking at the CD49f+ fraction of human cord blood progenitor cells, defined by the expression of cell-surface markers, with the SmartSeq2 protocol, suggested a continuous but polarised composition of the progenitor compartment. The data suggested a model of progressive lineage restrictions correlated with the expression of CLEC9A and CD34. Two progenitor cell subsets were identified, one committed to lymphoid lineage and the other multipotent and demonstrating long-term repopulating activity, thus suggesting that lineage restriction is already in place in the early progenitor compartment [26] which was hypothesised by earlier studies [19,21]. Collectively, these studies point towards a remarkable heterogeneity of the haematopoietic progenitor compartment, which was not previously appreciated.

### Towards a new model of the haematopoietic tree

Another important observation derived by these and other studies utilising single-cell technologies is that haematopoiesis is less of a stepwise process than previously suggested. As a consequence, the haematopoietic tree was revisited. An unbiased scRNA-seq analysis of haematopoietic cells from eight zebrafish transgenic reporter lines, each labelling a different cell type, was recently used to reconstruct the zebrafish blood lineage tree *in vivo*. The analysis, using the SmartSeq2 platform, demonstrated that progenitors undergo a gradual transcriptional transition from the multipotent state to lineage restriction and that there is a high cell-to-cell variability in the propensity of haematopoietic progenitors to differentiate down the separate lineages [27].

A recent study integrated single-cell transcriptomics with the QUARTZ-seq protocol and functional assays to characterise human healthy haematopoietic progenitors from the bone marrow. The analysis of the integrated data suggested that HSCs and Lin- CD34^+^ CD38^−^ cells form a continuum of low-primed undifferentiated haematopoietic progenitors (‘CLOUD’)-HSPCs, while the downstream part of the tree is dominated by unilineage-restricted progenitors that are associated with the up-regulated expression of CD38. This points towards a continuous acquisition of different lineage restrictions without going through the traditional hierarchy of multi-, oligo- and unipotent progenitors [28]. More recently, two studies confirmed this concept in the mouse. In the first, using the inDrop platform, researchers performed scRNA-seq on more than 4000 Kit+ blood progenitors derived from mouse adult bone marrow and coupled it with single-cell cultures and population balance analysis, a method used to predict cell fate from transcriptomics data. The analysis showed that progenitors fall on a continuum of cell states, which terminate into seven possible cell fates. While some cell fates were correlated, such as the erythroid and granulocytic ones, the analysis showed haematopoietic progenitors did not segregate into homogeneous states [29]. In the second study, the authors profiled 80 000 Kit+ cells from the mouse bone marrow by MARS-seq, combined with perturbation studies using CRISP-seq for transcription factors involved in myeloid lineage commitment. The analysis showed exclusive coexistence of myeloid and lymphoid and no erythroid gene expression signatures in the blood progenitor compartment. These data suggest that myeloid and lymphoid states form a network of possible cell differentiation fates which is not part of a hierarchical structure [30]. Another study recently looked at unperturbed haematopoiesis in mouse using the inDrops protocol combined with transposon-tagging tracing and suggested that, while phenotypic HSCs contribute mainly to the megakaryocyte lineage, the other myeloid and lymphoid lineages largely derive from a heterogeneous multipotent population, which contains a continuum of primed and unprimed states [31].

To summarise, the use of scRNA-seq moved the field of haematopoiesis forward by revisiting the traditional haematopoietic tree to reflect the newly appreciated level of heterogeneity in the progenitor compartment. The structure of the haematopoietic tree is however still subject to debate. Looking ahead, sharing scRNA-seq datasets and method scripts, as part of initiatives such as the Human Cell Atlas [32], will push the field forward by helping researchers to test different hypotheses and algorithms. In addition, the combination of single-cell multi-omics approaches will allow a better characterisation of blood progenitors and add new layers of understanding on the processes driving normal and diseased haematopoiesis.

## ScRNA-seq to reveal heterogeneity of immune system

To assure the high level of protection, immune cells possess incredible diversity and versatility. Advances in flow-cytometry and microscopy contributed to our understanding of different components of the immune system. However, the number of parameters that can be measured using these technologies is limited and requires prior knowledge of expressed cell-surface markers. Accurate classification of immune cell types is crucial to understand the functional configuration of the immune system. Therefore, single-cell technologies emerge as a powerful tool to fully uncover the complexity of the immune system. Single-cell transcriptomics has been successfully applied to discover new immune cell types and cell states [33,34], identify molecular pathways engaged in immune responses [35,36] and investigate lymphocyte antigen receptor diversity [37,38] (Figure 1). As the application of single-cell technologies in immunology is constantly expanding, it makes it almost impossible to cover all the relevant information in one paper. Therefore, we would like to refer the readers to other excellent reviews about single-cell approaches in the immunity context for more details [39–41].

### Discovery of new immune cell types

To ensure sufficient level of protection against pathogens and diseases, immune cells are characterised by extreme heterogeneity. ScRNA-seq is transforming our ability to characterise rare cell types, often overlooked in bulk population analysis. It gives a unique opportunity to study rare immune cells in their microanatomical niches. That has contributed to the discovery of new cell types and cellular states underlying the heterogeneity and plasticity of the immune system [42]. Two of many examples are the studies of Villani et al. [33] and Björklund et al. [43] in which application of single-cell transcriptomics allowed for reclassification of dendritic cells and monocytes populations from human blood and dissection of innate lymphoid cells (ILCs) heterogeneity from human tonsils.

However, single-cell transcriptomics contributed not only to redefining human immune landscape but also to exploring immune components of lower vertebrates, like zebrafish [34,44–46]. This is especially relevant as recently there has been an increased interest in fish immunology. For evolutionary biologists, fish immune system provides an important comparative outgroup for understanding the evolution of the immune system [47,48]. Application of scRNA-seq allowed to obtain and characterise first transcriptomes of zebrafish specific immune cell types (T cells, natural killer (NK) cells and myeloid-like cells). Differential expression analysis uncovered new immune cell-specific genes shedding more light on the evolution of immune system [45]. In other studies, single-cell transcriptomics revealed the existence of Th2 and regulatory T cells (Tregs) populations in zebrafish further confirming similarity in immunobiology between mammals and teleost fish [44,46]. Finally, application of 10x RNAseq, which allows to analyse thousands of cells in a single run, contributed to the discovery of rare innate lymphoid cells in zebrafish gut which so far has been described only in mammalian species [49]. In this study, single-cell transcriptomics not only contributed to the discovery of a new cell type but also shed light on transcriptome changes related to cell states in response to the immune challenge which demonstrated the power of single-cell transcriptomics to reveal plasticity of cell states [34].

### Dissecting molecular pathways of immune responses

Our understanding of mechanisms underlying immune response has important consequences for basic research as well as translational studies. Looking at the composition and trajectories of immune cells development in healthy and pathological environment can contribute to the identification of molecular drivers of disease and immune escape mechanisms, resulting in better understanding of generation and progression of human diseases. First step in achieving this goal is examining the tissue-specific signature in different microenvironments. Recent studies from Crinier et al. [35] and Miragaia et al. [36] addressed this issue. They used scRNA-seq to characterise organ-specific differences in transcriptome of Tregs [36] and NK cells [35]. Taking these findings a step further requires scrutinising disease-associated changes in transcriptome similar to the study carried out by Kinchen et al. [50], where dysregulation of intestinal niche related to colitis was reported. An example where single-cell transcriptomics can be applied in this context in the immunology field is studying the differentiation pathways of myeloid-derived suppressor cells (MDSCs). MDSCs are considered to be a heterogeneous population of immature myeloid cells with immunosuppressive phenotype which are involved in tumour escape mechanisms [51]. ScRNA-seq provides a unique platform for comparative analysis of developmental trajectory of normal and perturbed myelopoiesis with a view of identifying potential druggable pathways blocking generation and accumulation of MDSCs.

### Antigen receptor diversity at single-cell level

The specificity of adaptive immune response relies on the diverse repertoire of T-cell receptors (TCR) and B-cell receptors (BCR). These are antigen-specific receptors expressed on the surface of T and B lymphocytes. After encountering the antigen, T and B cells undergo clonal expansion. Single-cell transcriptomics has the power to combine information about adaptive lymphocyte clonal dynamics with transcriptional phenotype of cells to study adaptive immunity in great details and help unravel pathological mechanisms [37,52]. TraCeR and BraCeR are computational methods that have been recently developed for reconstructing antigen-receptor sequences from scRNA-seq data [37,38]. Other tools were also successfully created including scTCRseq [53], TRAPeS [54] and VDJ Puzzle [55]. Currently, 10x Genomics introduced Single Cell immune Profiling Solution, which will allow to map adaptive immune receptors repertoire and dissect their clonality at a greater scale. It is now possible to obtain simultaneous information about genes expression profile together with TCR/BCR sequence from the same cell. Studies investigating TCR and BCR clonality in the context of disease already contributed to understanding the expansion of specific lymphocyte clones in diseases such as cancer [56,57]. For example, analysis of TCRs clonality in patients with hepatocellular carcinoma (HCC) revealed clonal expansion of CD8^+^ specific to tumour tissues suggesting that local T cells proliferate in tumour-rich environments. Clonality analysis highlighting clonal expansion of exhausted CD8^+^ T cells in HCC microenvironment suggested another possible mechanism of tumour immune escape [52]. Similar study of breast tumour microenvironment showed that tumour-associated T-cell states are shaped by a combination of TCR usage and environmental stimuli [57].

All the above examples prove that single-cell profiling of immune cell dynamics has already substantially improved our knowledge of immune mechanisms. Further advances in single-cell transcriptomics combined with multidisciplinary research shedding light on immune mechanisms will allow for development of more precise vaccination and treatment strategies.

## Current challenges and future directions

ScRNA-seq revolutionised the way we study the haematopoietic and immune system. However, many limitations still persist. The first step of every scRNA-seq protocol is the generation of a single-cell suspension from the tissue of interest. This leads to two main issues. Firstly, in order to have a representative picture, the single-cell suspension generated has to closely reflect the cell composition of the original tissue. This can be a challenge as some cell types are more sensitive to enzymes used in dissociation protocols, thus leading to a biased proportion of cell types being analysed [58]. Secondly, there have been reports of altered gene expression patterns related to tissue processing [59].

The vast majority of technologies used for scRNA-seq only capture polyadenylated RNA, thus leaving out microRNAs and other regulatory RNAs, although new methods are now being developed to capture the total RNA content of an individual cell [60]. Another outstanding problem is the difficulty of functionally validating the subpopulations identified by clustering of scRNA-seq data. In addition, in currently used scRNA-seq technologies there is complete loss of data spatial contextualisation, which is essential for the characterisation of cell interactions and tissue structure. To overcome this problem, different laboratories are developing spatial transcriptomics methods, some of which rely on the superimposition of tissue sections on barcoded primers [61,62], while others are based on the *in situ* hybridisation method [63,64].

The rapid development of scRNA-seq technologies led to a parallel burst of computational methods for the analysis of the large dataset generated. The most utilised methods available were recently summarised by Hwang et al. [65]. One of the biggest challenges that still remain is the integration of datasets generated with different platforms. Overcoming this issue is of great importance as it will facilitate the interrogation and reuse of large datasets already available.

As technology progresses quickly, the application of scRNA-seq approaches will shift more from the descriptive analysis of tissue heterogeneity, which includes atlasing efforts, towards the understanding of the mechanisms involved in disease onset and progression, particularly in the fields of immunity and cancer. This has been reviewed in detail here [66]. Looking forward, complementing the analysis of the transcriptome with other ‘omics’ approaches will enable an unbiased characterisation of a single cell. Combining scRNA-seq and DNA sequencing, for example, will allow the reconstruction of developmental lineage trees, while integrating the analysis of the transcriptome with that of the epigenome will elucidate mechanisms of regulation of gene expression at the whole-genome scale [67].

## Summary

- Application of scRNA-seq moved the field of haematopoiesis forward by revisiting the traditional haematopoietic tree to reflect the newly appreciated level of heterogeneity in the progenitor compartment.
- Single-cell transcriptomics revolutionised our understanding of immune system by revealing the plasticity of immune cells and mechanisms of immune responses at high resolution.
- Main limitations of scRNA-seq include: focus on polyadenylated RNA, gene expression changes related to tissue processing, loss of spatial context and functional validation.
- Further integration of single-cell transcriptomics with other omics approaches will expand our knowledge in haematopoiesis and immunology.

## Abbreviations

BCR: B-cell receptor
HCC: hepatocellular carcinoma
HSC: haematopoietic stem cell
HSPCs: haematopoietic stem and progenitor cells
ILC: innate lymphoid cell
MARS-seq: massively parallel single cell RNA sequencing
MDSC: myeloid-derived suppressor cell
NK cell: natural killer cell
scRNA-seq: single-cell RNA sequencing
STRT-seq: single-cell tagged reverse transcription sequencing
TCR: T-cell receptor
Treg: T regulatory cell
